# Giant Amoebic Liver Abscess: A Rare Diagnosis in a Rural Setting of Sub-Saharan Africa

**DOI:** 10.1155/2021/2825778

**Published:** 2021-10-19

**Authors:** Cyril Jabea Ekabe, Jules Kehbila, Njinju Asaba Clinton

**Affiliations:** ^1^Grace Community Health and Development Association, P. O. Box, 15 Kumba, Southwest Region, Cameroon; ^2^Mbonge District Hospital, South West Region, Cameroon; ^3^Faculty of Health Sciences, University of Buea, Cameroon; ^4^Wum District Hospital, Wum, Northwest Region, Cameroon; ^5^Health and Empowerment Foundation, , Cameroon

## Abstract

**Background:**

Extraintestinal amoebiasis is an uncommon complication of *Entamoeba histolytica* infection, occurring in about 5-10% of patient. Prompt diagnosis and management is essential to prevent complications. However, diagnosis and management in resource-limited settings is very challenging owing to limited diagnostic tools and nonspecific clinical symptoms. Therefore, our case report underscores the role of incisive clinical evaluation, basic investigation, and nonsurgical management of giant amoebic abscess in resource-limited settings. *Case Presentation*. A 13-year-old female Cameroonian presented with subacute onset of upper abdominal pain, high fever, and chest pain for one week. Before presentation, she had been on treatment at a local traditional practitioner during which her symptoms worsen. After clinical evaluation and basic investigation, she was diagnosed with a giant amoebic liver abscess. She was resuscitated and placed on nonsurgical management. Follow-up after 1 month was significant for complete recovery.

**Conclusion:**

Amoebic liver abscess is a rare complication of *Entamoeba histolytica* infection with devastating complications. The diagnosis of this disease requires high index of suspicion in resource-limited settings. Good clinical evaluation and timely nonsurgical therapy can provide recovery to some patients.

## 1. Background

### 1.1. Introduction

*Entamoeba histolytica* is an enteric protozoa parasite and the causative agent of amoebiasis. Worldwide, there are over 50 million cases and 40,000-100,000 associated deaths. It is ranked as the second leading cause of death from intestinal parasitic infections and the third cause of parasite-related mortality after malaria and schistosomiasis [1]. *E. histolytica* exists in three forms: the trophozoites or vegetative form, the precyst, and the cyst. The infective stage is the quadrinucleated mature cyst of the protozoa, which is passed in feces from infected individuals or carriers [2].

**Figure 1:**
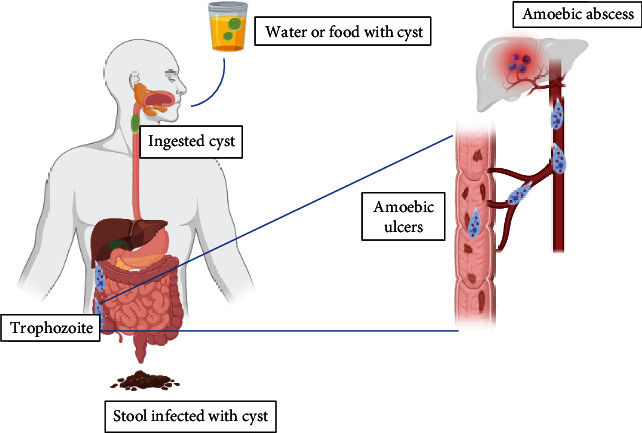
A diagram illustrating a summary of the life cycle *E. histolytica* and the spread of trophozoites to the liver through the portal circulation.

Infection is acquired via ingestion of mature quadrinucleated cyst in contaminated food or water. These matured cysts are capable of living in the external environment for up to 10 days and are resistant to gastric acid and unfavorable environmental conditions because of their thick walls. After ingestion, the cyst undergoes excystation at the lower part of the ileum and caecum in response to increase alkalinity and trypsin-induced damage of the cyst wall. Following the process of excystation, eight metacystic motile trophozoites are released, which eventually migrate to the large intestine. In the small intestine, the trophozoites undergo binary fission to form cyst which is later passed out in feces. Trophozoites pass out in feces are usually not infective, because they are killed by heat, dryness, and other environmental conditions [3, 4]. The life cycle of *E. histolytica* is summarized in Figure 1 shown below.

In a majority of cases, the infection is asymptomatic. Many hypotheses have been postulated to explain why certain patients have asymptomatic disease, while others progress to invasive disease. Some identified factors like strain virulence, environment, and the host's genetic susceptibility, immune status, age, and gender have all been found to predict disease severity [5]. The pathogenesis of amoebiasis is mediated by binding of the trophozoites to the specific receptors expressed in the large intestinal epithelium. These metacyclic trophozoites express Gal/GalNAc lectin which binds to galactose and N-acetyl-D-galactosamine residues found on O-linked sugar side chains of mucins [6, 7]. Mammals lacking the N terminal galactose or N-acetyl-D-galactosamine are resistant to trophozoite adherence, providing some degree of immunity against invasive disease [8]. The colon contains higher number of mucus cells than the small intestine; hence, this could be a potential reason for tropism of the trophozoites to this area. One of the key virulence factors of *E. histolytica* is the production of mucin breaking enzymes (glycosidases such as sialidase, N-acetylgalactosaminidase, N-acetylglucosamines, and cysteine proteases) that destroy the columnar epithelium of the crypts of Lieberkühn of the large intestine, promoting penetration, and formation of flask shaped ulcers. Other mechanisms involve apoptosis, inflammation, and formation of amoebapores causing cytolysis of infected cells. In some individuals, the trophozoites can break through the intestinal wall into the peritoneum and portal circulation, where they migrate to the liver, lung, and other extraintestinal sites causing severe disease [1].

## 2. Clinical Presentation

*Entamoeba histolytica* causes amoebiasis, which is seen clinically in about 10% those infected [2]. Amoebiasis is classified into intestinal and extraintestinal amoebiasis. Amoebic colitis is the most common presentation of intestinal amoebiasis. It has a subacute onset and presents with nonspecific symptoms such as watery diarrhoea, abdominal discomfort, and severe dysentery [9]. Amoebic colitis might progress to fulminant necrotizing colitis, toxic megacolon, fistulating ulcers, amoeboma formation, and peritonitis [10]. Amoebomas are granulomatous swellings which uncommonly occur in amoebic colitis. They typically occur in the cecum and ascending colon and might be mistaken for lymphoma or neoplasms. Amoeboma usually presents with right iliac fossa pain or obstructive symptoms [11].

The most common presentations of extraintestinal amoebiasis are amoebic liver abscess (ALA) and pulmonary amoebiasis. Amoebic liver abscess occurs in 5-10% of individuals with amoebiasis. Above 50% of individuals present with fever and constant arching right upper quadrant pain within 2-4 weeks of infection. Other symptoms include weight loss, diarrhoea, cough, and pleural effusion [12, 13]. Fever, abdominal tenderness, and hepatomegaly are the most common epidemiological characteristics of ALA in children, as shown in Table 1 [14].

Additionally, important laboratory findings in ALA include anemia, leukocytosis, and increased transaminases. Ultrasound investigations usually reveal the presence of a solitary abscess, although scarcely multiple small abscesses may occur. Some complications of amoebic liver abscess include pulmonary amoebiasis and rupture of the abscess into the pericardial cavity [15].

Pulmonary amoebiasis is the second most common form of extraintestinal amoebiasis. It usually occurs as a result direct extension of amoebic liver abscess or through hematogenous or lymphatic spread from the loci of infection. The right lower or middle lobe of the lung is most commonly affected. Common clinical symptoms include fever, hemoptysis, right upper quadrant pain, and referred pain to the right shoulder or intrascapular region. Pulmonary abscesses, bronchohepatic fistula, and empyema can occur with rupture of liver abscess [16].

### 2.1. Diagnostic Test for *E. histolytica* and Amoebic Liver Abscess

Several laboratory techniques have been developed for the diagnosis of amoebiasis. Some of these include serological assays, parasitological tests (microscopy and culture), and the highly sensitive molecular techniques such as PCR-based assays. In remote settings, microscopic examination of stool samples for the presence of characteristic cyst or trophozoites remains the gold standard. Stool microscopy plays a vital role in the diagnosis of amoebiasis. The presence of *E. histolytica* is confirmed by its characteristic small mature cyst containing four nuclei [17].

The sensitivity of microscopy is poor and can be confounded with false positive results such as the identification of macrophages as trophozoites or polymorphonuclear cells as cysts [18]. Furthermore, microscopy remains less reliable to distinguish between *Entamoeba* species when compared to other diagnostic methods such as culture or antigen detection tests [19].

Stool culture can be used to diagnose *E. histolytica*. The main technique is the xenic culture of rectal biopsy specimens, fecal specimens, and liver aspirates [20–22]. Despite the ability of the culture method to distinguish between the different types of *Entamoeba* species, this procedure turns to be difficult, expensive, very prone to bacteria contamination, and labor-intensive to maintain in the laboratory [21]. Serological tests are very vital in the diagnosis of amoebic liver abscess where detection of parasites in feces might not be feasible. These include indirect hemagglutination assay (IHA), latex agglutination, the amoebic gel diffusion test, immunodiffusion, complement fixation, indirect immunofluorescence assay (IFA), and enzyme-linked immunosorbent assay (ELISA) [17]. The serological assays remain a problem in areas where amoebiasis is endemic and people are continuously being exposed to *E. histolytica*. This is due to the inability of the assay to distinguish past from current infection, thus making it difficult to arrive to a definitive diagnosis [23]. Other laboratory investigations include molecular laboratory test like PCR-based assays. The higher sensitivity of this molecular technique over the parasitological techniques makes it more suitable for diagnosing disease conditions with very low parasite load [24, 25].

Basic imaging techniques like ultrasound are indispensable for the diagnosis of ALA. It has an advantage of being not noninvasive and relatively cheap. Ultrasonography has a high accuracy rate of over 95% and has been considered the gold standard imaging technique for the diagnosis of ALA. Common ultrasound findings in amoebic liver abscess are a solitary hypoechoic mass with a wall, containing debris or necrotic material. The abscesses are usually located in the posterosuperior surface of the right lobe than the left lobe [24–28]. The use of CT scans is less important compared to the ultrasonography for the diagnosis of ALA [26, 27]. Chest X-ray investigations are usually indicated for detection of pleural and pericardial effusions [24, 25, 28].

### 2.2. Treatment of *E. histolytica* and Amoebic Liver Abscess

Treatment measures of amoebiasis include pharmacologic therapy, surgical intervention, and preventive measures. For mild disease, an outpatient treatment is employed, but severe and invasive disease states like severe colitis, liver abscess, and hypovolemia require intravenous (IV) volume replacement and empiric therapy; fulminant colitis requires surgical evaluation; peritonitis and suspected amoebic liver abscess rupture will require hospitalization [29]. Asymptomatic infections are not treated in nonendemic areas; nonetheless, intraluminal drugs like paromomycin, iodoquinol, and diloxanide furoate are recommended for treatment [30–32]. This is done to prevent invasive infections and reduce cyst shedding into the environment [33]. Nitroimidazoles, particularly metronidazole, are the mainstay of therapy for invasive amoebiasis [29, 30, 34–36]. Moreover, nitroimidazoles with longer half-lives (namely, tinidazole, secnidazole, and ornidazole) are better tolerated and allow shorter periods of treatment but are not available in many countries. Since these drugs do not affect intraluminal parasites, luminal agents (paromomycin or diloxanide furoate) are used to prevent relapse following treatment of amoebic colitis [33]. Recent Cochrane Database Review reports that tinidazole may be more effective than metronidazole and is associated with fewer adverse events, and combination drug therapy may be more effective for reducing parasitological failure compared with metronidazole alone [30]. In case of bacterial super infection or suspected perforation broad spectrum, antibiotics are added to the treatment [29].

The treatment of ALA depends on its size; abscesses up to 10 cm can be managed with metronidazole without drainage [37], with clinical recovery in over 90% of cases [33, 38]. Surgical intervention is reserved for acute abdomen (perforated amoebic colitis, massive gastrointestinal bleeding, and toxic mega colon) [39], whereas drainage is reserved for larger abscesses, those at risk of rupture (>5 cm) and patients who do not response to medical therapy by about 3-5 days, or for left-lobe abscesses, which could rupture into the pericardium [33, 40, 41]. Drainage via image-guided percutaneous intervention (needle aspiration or catheter drainage) has replaced surgical intervention as the procedure of choice. Furthermore, comparative studies show that percutaneous catheter drainage is more effective in management of large amoebic liver abscess than needle aspiration [42, 43]. A luminal agent should also follow this treatment [33]. Preventive measures in the management of amoebiasis include food and water hygiene (boil water and wash fruits and food thoroughly), avoiding oral and anal sex, screening those exposed and those at risk, and development of a vaccine (which is still at its infancy stage) [44–46]. Our case report elaborates the importance of good clinical skills and nonsurgical management of giant amoebic liver abscess in rural communities with unsophisticated health care delivery facilities.

## 3. Case Report

A thirteen-year-old sub-Saharan female presented in a small community clinic with insidious onset of right hypochondriac abdominal pain and intermittent high fever, progressing over a one-month period. These symptoms began as dull, arching constant pain on the right hypochondria, radiating to the upper right scapula. The symptoms progressed with increased intermitted fever, dry cough, severe pleuritic chest pain, and mild breathing difficulties within one week before consultation. These symptoms were associated with anorexia, weight loss, body weakness, vomiting, and nonbloody mucoid stools. There was no jaundice, limb swelling, changes in skin color, or delusion. There were no other children with similar symptoms, and her past medical history was unremarkable for human immunodeficiency virus (HIV) infection, liver diseases, diabetes, syphilis, liver disease, renal disease, or abdominal trauma. Her travelling history is unremarkable. She is a pupil living a war risk rural community in Cameroon. Their main source of drinking water is stream water, and hygiene and sanitation in the community is poor. She had been on traditional herbs with scarification treatments at a local traditional doctor for three weeks, during which her symptoms progressively worsen. No previous medical consultations had been done.

On examination, she was pale, ill looking, and febrile with signs of respiratory distress, dehydration, and weight loss. Her conjunctivae were pale and sclera anicteric. She had a blood pressure of 90/60 mmHg, pulse was regular, fast, weak, and thready with a rate of 120 beats/min, respiratory rate is 32 breaths/min, temperature was 39 degrees Celsius, GCS is 15/15, and capillary refill > 2 seconds.

On cardiorespiratory examination, the heart rate was 120 beats per min, with normal first and second heart sounds. Examination of the respiratory system revealed decreased lung expansion on the right lower posterior lung, with stony dull percussion note, decreased lung sounds, and decreased vocal fremitus, with fine crackles. Examination of other lung fields is normal.

Examination of the abdomen revealed a distended abdomen with tenderness on deep palpation of the right and left upper abdominal quadrants. Abdominal palpation was significant of massive tender firm hepatomegaly measuring 12 × 10 cm, with smooth surface and mild splenomegaly. Examination of other quadrants was normal. Normal bowel sounds were present. All other exams were unremarkable. Based on her presentation, we suspected liver abscess, community-acquired pneumonia, hydatid cyst, hepatic adenomas, and traumatic liver injury.

**Figure 2:**
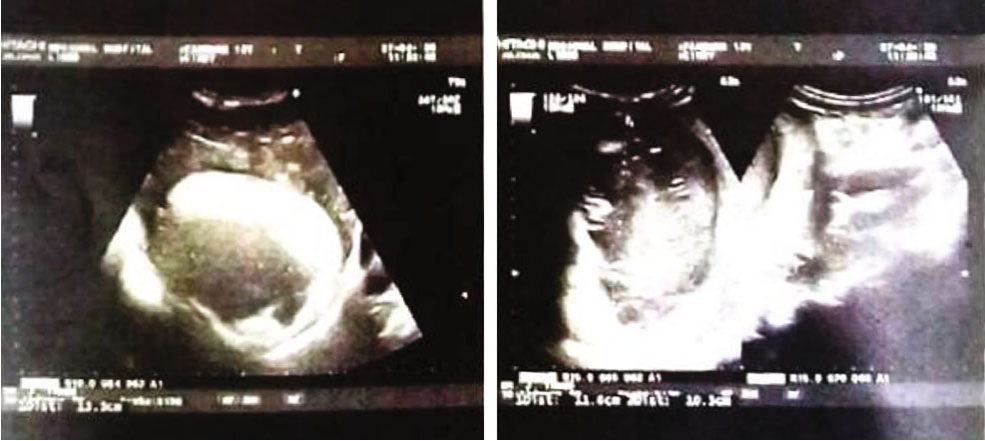
The ultrasound reveals a giant heterogenous mass with thick walls and air bubbles within the right lower lobe of the liver measuring 141 mm suggestive of an amoebic liver abscess.

She was immediately resuscitated with normal saline (0.9%), and blood, stool, and urine samples were collected for laboratory analysis. An abdominal echography and chest X-ray were also requested. The analyses revealed a hemoglobin of 8 g/dl, WBC 25,000 cells/*μ*l, HIV test: negative, and malaria RDT test: negative. Stool exam reveals the presence of Charcot Leydig crystals and *E. histolytica* cyst. Other findings were normal. The results of the abdominal ultrasound revealed a huge heterogenous mass within the right lobe of the liver with thick walls, air bubbles, and a dimension of 141 mm suggestive of amoebic liver abscess (Figure 2). In addition to abdominal ultrasound examination, the chest X-ray reveals pleural effusion on the right lower left lung.

Grounded on the clinical presentation and findings on investigation (ultrasound and stool exam), we made a diagnosis of a giant amoebic liver abscess with pleural effusion. According to standard indications, we referred the patient to a surgeon in a referral hospital. However, because of financial constraints, they could not meet up with the demands. To save the patient, the community hospital decided to embark on a nonsurgical treatment of the patient sponsored by a local nongovernmental organization (GRACHADA). The patient was placed on metronidazole infusion (500 mg thrice daily for one week), 0.9% normal saline (1.5 liters daily for 5 days), ceftriaxone 2 grams daily, glucose 5% (1 liter daily for 5 days), and prednisolone 5 mg (10 mg thrice daily for 7 days). After 7 days of treatment, there was marked clinical improvement of the patient. Vital signs were stable, and serial evaluation with abdominal echography showed decrease in the size of the abscess with decreased heterogenicity. The patient was discharged and relayed on oral treatment with metronidazole (for two weeks), prednisolone (5 mg twice daily for two weeks), and protein energy supplements for 2 weeks. Follow-up of patient after two weeks was remarkable for full recovery of patient, with resolution of abscess on echography.

## 4. Conclusions

Amoebic liver abscess is the most common extraintestinal manifestation of amoebiasis presenting in approximately 5-10% of patients with amoebiasis. ALA is linked with complication like pulmonary amoebiasis. Early diagnosis of ALA above 100 mm is critical, as this has been shown to be associated with high risk of rupture. Rupture of ALA can cause fatal consequences like peritonitis, empyema, and cardiac tamponade. The pathogenesis of ALA is as a consequence of hematogenous spread of trophozoites from the intestinal loci through the hepatic portal vein in the liver. The diagnosis of the ALA requires a high index of suspicion especially because of the presence of nonspecific symptoms and similarity to other liver pathologies that present with hepatomegaly. Nevertheless, like in our case, an astute history and physical examination can throw more light into the diagnosis of the disease. Furthermore, in resource-limited communities like ours where advanced diagnostic techniques are absent and health care delivery is substandard, health personnel can only rely on good clinical history and examination and basic diagnostic techniques to diagnose this disease. The presence of progressive fever and right upper quadrant pain in the context of poor hygiene and sanitary background increased our suspicion of ALA. However, these symptoms were nonspecific and necessitated more investigation.

Basic laboratory investigations like full blood count and stool exam are fundamental in the diagnosis of this pathology, as evident in our case. The occurrences of leukocytosis and *E. histolytica* cysts in stool were imperative findings. Nevertheless, the presence of leukocytosis and other inflammatory markers is nonspecific as this can be associated with other infections. As described above, the detection of either the trophozoites or quadrinucleated cyst is definitive for diagnosis of amoebiasis. However, various laboratory precautions are necessary for appropriate detection of the parasite. For example, because of the susceptibility of trophozoites to harsh external conditions, early analyses of collected stool sample are pivotal for trophozoite detection. Also, it is advisable to collect at least three stool samples for appropriate analyses for trophozoites and cyst. In our case, three morning samples of stool were collected for analyses which revealed the presence of cyst and Charcot Leydig crystals. This increased our suspicion of ALA, although cyst detection is absent in some cases [47]. To buttress our suspicion, an ultrasound was indicated. Ultrasonography has a high accuracy rate of over 95% and has been considered the gold standard imaging technique for the diagnosis of ALA [24, 25, 28]. This was utilized to confirm the diagnosis of ALA in our patient. However, ultrasound-guided aspiration was not exploited in our case, because of lack of resources. Ultrasound-guided aspiration and biopsy would have been important for laboratory analyses and diagnosis of ALA in our patient. However, because of limited access to facilities necessary to carry out such procedures and to manage fatal complication like bleeding, it was not done. Nevertheless, this was one of the reasons we decided to write on this case to show that with limited facilities, health professionals in resource limited can still do well to diagnose ALA. Another important finding was the presence of right lower pleural effusion, a common manifestation of pulmonary amoebiasis. Pulmonary amoebiasis has been postulated to result from direct extension of ALA in 35% of patients [14]. It presents with right lower pleural effusion, as evident in our case. Advanced techniques like PCR and ELISA for analysis of pleural fluid could not be exploited in our setting. ALA with dimensions greater than 10 cm is indicative for surgical intervention [37]. In our case, the diameter of ALA was 14 cm, hence necessitating surgery because of increased risk of rupture. However, because of financial constraints, a nonsurgical therapeutic intervention with antibiotics was tried for one week, which showed immense clinical improvement and recovery. Steroid therapy was also used to reduce the risk of hyperinflammation and improve recovery. This is probably because steroids have been shown to inhibit Th1 and response and improve Th2 immune response. Th2 immune response increases polarization of M2 macrophages important for tissue repair. Treatment was continued for three weeks, and follow-up echography revealed complete recovery with few signs of fibrotic tissues. This case emphasizes the importance of increased index of suspicion of ALA in patients presenting with fever and right upper quadrant pain, living in rural settings. It also underscores the plausible role of basic investigations like peripheral blood smear, stool exam, and ultrasound and the vitality of nonsurgical therapeutic trials in massive ALA for the time management in resource-limited settings. Although the role of steroids is not clearly defined in the management ALA, from our patient, we can infer that steroids are vital for good clinical recovery in patients with large amoebic abscess. Nevertheless, the role of steroids in the clinical recovery of patients with giant amoebic liver abscess needs to be investigated. This case exemplifies the importance of high index of suspicion and basic investigation in the diagnosis of ALA in resource-limited settings. Furthermore, timely nonsurgical management of ALA plays an important role in reducing the risk of ALA-related morbidity and mortality in communities with poor access to sophisticated health care.

## Figures and Tables

**Table 1:** Clinical and epidemiological characteristics of amoebic liver abscess.

Characteristics	Percentage of patients affected (%)
Fever	85–90
Weight loss	33–50
Diarrhoea	20–33
Cough	10–30
Jaundice	6–10
Abdominal tenderness	84–90
Hepatomegaly	30–50
Prevalence (male/female)	50/50 in children
Symptoms > 4 weeks	21–51

## Data Availability

All data are included within the article.

## Abbreviations

ALA: Amoebic liver abscess
ELISA: Enzyme-linked immunosorbent assay
HIV: Human immunodeficiency virus
WBC: White blood cells.
